# Supplementary material for: Relationship between School Backpacks and Musculoskeletal Pain in Children 8 to 10 Years of Age: An Observational, Cross-Sectional and Analytical Study
Source: Int J Environ Res Public Health. 2020 Apr 5;17(7):2487. doi: 10.3390/ijerph17072487 (PMC7177975; doi:10.3390/ijerph17072487)
Supplement: Supplementary file 1 [file ijerph-17-02487-s001.pdf]

Supplementary File S1

Data sheet of the participants

Part 1: Personal information

ID: CHIPAIN _

Gender:

- Boy
- Girl

Birth date:

School year:

Student weight:

Student size:

BMI:

Backpack weight:

Disorders associated with the spine:

Part 2: Related to the use of the backpack

1. How do you go to school?

- Transportation
- Walk

2. If you walk, how long does it take to get from home to school?

- <5 min
- 5-10 min
- 11-15 min
- 16-20 min
- 21-30 min
- >30 min

3. Do you carry your own backpack?

- Yes
- No

4. How do you normally carry the backpack?

- On both shoulders
- On one shoulder
- In my hand

5. Do you feel tired carrying your backpack?

- Never
- Almost never
- Sometimes

- Frequently
- Always

6. Do you think your backpack is too heavy for you?

- Yes
- No

7. When you arrive at school, do you leave the material that you bring in your backpack in the spaces dedicated to it?

- Yes
- No

8. Do your parents experience back pain?

- Yes
- No

9. How many hours do you spend watching TV or playing video games during the day?

- 0
- 1
- 2
- 3
- 4
- >5

10. What physical activities do you do?

- Physical education classes
- Other sports:

11. How many hours do you dedicate to extracurricular sports activities on a weekly basis?

- 0
- 1
- 2
- 3
- 4
- >5

If your back hurts and you have not had any injuries that cause this pain, please answer the following questions:

12. How often does your back hurt?

- Frequently (4-5 days/week or more)
- Sometimes (2-3 days/week)
- Occasionally (1day/week)
- Rarely (<1 day/week)

13. How long has it been hurting?

- <1 month
- 1-3 months
- 3-6 months
- 6 or more

14. How long does the back pain last?

- Less than 2 hours
- between 2 and 12 hours
- more than 12 hour

15. Have you missed school, other classes or sports because of pain?

- Yes
- No

16. Have you been to the doctor for this pain?

- Yes
- No

17. The pain gets worse if:

- You carry your backpack
- You lift the backpack
- You run
- It does not get wors
